# Properties of halogenated and sulfonated porphyrins relevant for the selection of photosensitizers in anticancer and antimicrobial therapies

**DOI:** 10.1371/journal.pone.0185984

**Published:** 2017-10-10

**Authors:** Barbara Pucelik, Robert Paczyński, Grzegorz Dubin, Mariette M. Pereira, Luis G. Arnaut, Janusz M. Dąbrowski

**Affiliations:** 1 Faculty of Chemistry, Jagiellonian University, Gronostajowa 2, Krakow, Poland; 2 Faculty of Biochemistry, Biophysics and Biotechnology, Jagiellonian University, Krakow, Poland; 3 Malopolska Centre of Biotechnology, Jagiellonian University, Krakow, Poland; 4 Chemistry Department, University of Coimbra, Coimbra, Portugal; Massachusetts General Hospital, UNITED STATES

## Abstract

The impact of substituents on the photochemical and biological properties of tetraphenylporphyrin-based photosensitizers for photodynamic therapy of cancer (PDT) as well as photodynamic inactivation of microorganisms (PDI) was examined. Spectroscopic and physicochemical properties were related with therapeutic efficacy in PDT of cancer and PDI of microbial cells *in vitro*. Less polar halogenated, sulfonamide porphyrins were most readily taken up by cells compared to hydrophilic and anionic porphyrins. The uptake and PDT of a hydrophilic porphyrin was significantly enhanced with incorporation in polymeric micelles (Pluronic L121). Photodynamic inactivation studies were performed against Gram-positive (*S*. *aureus*, *E*. *faecalis*), Gram-negative bacteria (*E*. *coli*, *P*. *aeruginosa*, *S*. *marcescens*) and fungal yeast (*C*. *albicans*). We observed a 6 logs reduction of *S*. *aureus* after irradiation (10 J/cm^2^) in the presence of 20 μM of hydrophilic porphyrin, but this was not improved with incorporation in Pluronic L121. A 2–3 logs reduction was obtained for *E*. *coli* using similar doses, and a decrease of 3–4 logs was achieved for *C*. *albicans*. Rational substitution of tetraphenylporphyrins improves their photodynamic properties and informs on strategies to obtain photosensitizers for efficient PDT and PDI. However, the design of the photosensitizers must be accompanied by the development of tailored drug formulations.

## Introduction

Photodynamic therapy (PDT) is a noninvasive and promising cancer treatment modality and has attracted considerable attention in recent years [1]. Properly designed photosensitizer (PS) is crucial for the final outcome of PDT. Modified tetrapyrrolic-based photosensitizers are highly attractive phototherapeutic agents not only for PDT of cancer, but also for photodynamic inactivation of microorganisms (PDI). PDI has recently been considered as a viable alternative method to antibiotic chemotherapy of infective diseases [2, 3]. PDT is based on the generation of reactive oxygen species (ROS) in the biological target by a combination of light, a photosensitizer and molecular oxygen. The broad range of photosensitizers employed in PDT and PDI includes naturally occurring and synthetic compounds modified by various substitution patterns [4, 5]. Current progress in the development of photosensitizers relies on the identification of specific design parameters. On one hand, there is an increasing appreciation of the need to integrate in molecular design insights emerging from studies of PS-target interactions, cellular signaling processes and increased resistance [6, 7]. On the other hand, photophysical and photochemical properties of PS can be related with its behavior in biological systems, namely with aggregation, tumor type-specific PS uptake mechanisms, binding to plasma proteins or change in hydrophilicity/lipophilicity in given physiological environments.

It is tempting to transfer the knowledge from anticancer PDT to PDI of microorganisms. However, several differences in the PS properties adequate for each of these applications must be highlighted. While PDT agents must strongly absorb in the phototherapeutic window, this is not as critical for antimicrobial approaches. The application of blue light for infectious diseases is well documented. Photons from this region may lead to potential biomedical applications (the treatment of acne vulgaris which is an important dermatologic disorder) as well as environmental fields such as water disinfection, decontamination systems for air, contact surfaces, and medical instruments. The presence of a positive charge in the PS structure seems to be important to target Gram-negative bacteria [8, 9], but negatively charged molecules may be effective for Gram-positive bacteria. Moreover, they seem to be more selective against bacteria than mammalian cells because they do not cross cell membranes efficiently. As the phototherapeutic agents are indiscriminate in their action on bacterial and host cells, it is imperative that PS is preferentially directed to bacterial cells rather than host cells before activation with light. Photosensitizers for PDI of microorganisms that are positively-charged (innate or modified by cationic molecular vehicles *i*.*e*. poly-L-lysine) porphyrin derivatives tend to have a superior activity towards microbial species and a relative selectivity over host mammalian cells, enabled by increased binding and penetration through the negatively charged bacteria outer barrier [2, 10–12]. The ability of the cytotoxic agents to preferentially target bacteria over healthy mammalian cells can be also achieved by specific conjugation with antimicrobial peptides [13]. Interesting biological properties have also been reported for various non-tetrapyrrolic compounds with different molecular frameworks [14, 15], such as functionalized fullerenes that act not only as highly effective PDI agents but also as antiretroviral dyes in cells, or benzo[*a*]phenoxazine chalcogen analogues that were proposed as broad-spectrum antimicrobial agents [16–18].

Tetrapyrrolic derivatives remain the most promising PDT photosensitizers in terms of strong absorption in the visible and excellent photosensitizing ability due to their long-lived triplet states. The PS lowest-electronically excited triplet state is the precursor of reactive oxygen species (ROS), which are the major cytotoxic agents during photodynamic action. They are generated either by transferring an electron/hydrogen atom (Type I processes) or electronic energy (Type II processes) to molecular oxygen, with the formation of oxygen-centered radicals (e.g. superoxide ion and hydroxyl radicals) or singlet oxygen, respectively [19, 20]. Rational molecular design of PS can determine many of their photophysical properties. For instance, protonation or incorporation of metal ions or halogen atoms in the macrocycle changes the balance between fluorescence and intersystem crossing and can influence many photophysical parameters [21–24]. It is generally recognized that molecules bearing fluorine, chlorine or other halogen atoms display enhanced photoinduced cytotoxic properties [25–27]. This is assigned to the “heavy atom effect” resulting from the increased spin-orbit coupling in the presence of atoms with higher atomic number and, consequently, higher spin-orbit coupling constants that enable efficient intersystem crossing, large triplet quantum yields and the generation of ROS with high yields [16, 28]. We have recently observed that for some chlorinated tetrapyrroles the heavy atom effect is so enhanced that can lead to almost complete loss of the fluorescent properties of PS and makes it much more difficult to study by fluorescence-based techniques [20]. Another important issue in designing efficient photosensitizers is their lipophilicity and solubility in biocompatible media. It is widely recognized that the hydrophobic nature of the majority of unmodified porphyrins is a limiting factor for their biomedical applications. Thus, hydrophilic substituents are frequently inserted into the macrocycle in order to develop clinically relevant photosensitizers [29]. Various hydrophilic substituents have been introduced in the phenyl rings of tetraphenylporphyrin (TPP) and their effects on the photodynamic behavior of the compounds have been examined. The number of polar groups and pattern of the substitutions significantly affect the photocytotoxicity of porphyrin derivatives and phthalocyanines [24, 30–32]. Another strategy for improved drug delivery is based on the use of specific carriers such as nanoparticles, liposomes or micellar formulations [33]. Among them, polymeric micelles are renowned for modulating the drug efficacy thanks to targeted delivery and effective solubilization of hydrophobic molecules [34–36]. PEG-conjugated photosensitizers, other micellar modification of tetrapyrroles as well as various hybrid materials have also been synthesized and examined *in vitro* and *in vivo* for PDT applications [37, 38]. The rational design of PS for PDI of microorganisms has been mostly based on the modification of porphyrins [5, 39].

Herein, we report a series of synthetic halogenated porphyrin derivatives as multifunctional photosensitizers for biomedical applications. In particular, we discuss the effect of peripheral substituents in the macrocycle on the photophysics, photochemistry and *in vitro* photodynamic efficacy with respect to biological targets (cancer cells and microbes). The molecular design involves peripheral substituents to obtain polarity-tunable compounds. Furthermore, halogen atoms (-F, -Cl) in the *ortho* positions of the phenyl rings in the *meso* position of macrocycle improve the photophysical properties by promoting intersystem crossing and increase singlet oxygen quantum yields. The effects of the number of substituents and major substitution pattern are discussed in the context of the hydrophobicity, the relative efficiency of ROS generation and overall *in vitro* photocytotoxicity. It is shown that the photodynamic activity of these PS is related to their molecular structures and drug formulations. We show that porphyrin derivatives offer a very convenient template to design and test phototherapeutic agents for PDT and PDI.

## Materials and methods

### Spectroscopic measurements

All commercial chemicals and reagents were of analytical grade and were purchased from Sigma-Aldrich. UV/Vis absorption spectra were recorded in a Hewlett Packard HP8453 spectrophotometer. Solutions containing samples of photosensitizers were dissolved in the selected solvent in quartz cuvettes. Using measured absorbance for various concentrations of porphyrin in ethanol, the molar absorption coefficients were determined from Beer’s law.

Fluorescence emission spectra were recorded from 550 nm to 750 nm with excitation at Soret band (λ≈420 nm). The excitation and emission slits were both set to 8 nm and scanning speed to 50 nm/min. Fluorescence spectra were recorded with a Perkin Elmer Fluorescence Spectrometer LS 55. Determination of fluorescence quantum yields: Φ_F_ of studied photosensitizers was determined using the comparative method according to the equation:

$$\Phi_{F}=\Phi_{FStd}\cdot \frac{FA_{Std}n^{2}}{F_{Std}An_{Std}^{2}},$$ (1)

where F and F_Std_ are the areas under the fluorescence emission curves of the sample and the standard, A and A_Std_ are the absorbance of the sample and the standard and n are the refractive indices of the solvent used for the sample and the standard, respectively. Commercially available tetraphenylporphyrin TPP (Std) (Φ_F_ = 0.10 in toluene) was employed as the standard. The absorbance of the solutions at the excitation wavelength was in the range of 0.02. Singlet oxygen quantum yields were determined based on the relative phosphorescence emissions of singlet oxygen generated by a reference and by each one of the porphyrins, using a method described elsewhere [19]. Briefly, a modified Applied Photophysics LKS.60 flash photolysis spectrometer with a Hamamatsu NIR photomultiplier for detection and HP Infinium (500 MHz, 1GSas^-1^) or Tektronix DPO 7254 (2.5 GHz, 40GSas^-1^) oscilloscopes were used. The adaptation of this spectrometer allowed for the detection of singlet oxygen phosphorescence at room temperature. This emission was detected using a Hamamatsu R5509–42 photomultiplier, cooled to 193 K in a liquid nitrogen chamber. Excitation was achieved with the third harmonic of Nd:YAG lasers (Spectra-Physics Quanta Ray GCR 130, 5–6 ns FWHM, or EKSPLA PL 2143 A, 30 ps pulse width). The modification of the spectrometer for time-resolved singlet oxygen phosphorescence measurements involved the interposition of a Melles Griot cold mirror (03MCS005), which reflects more than 99% of the incident light in the 400–700 nm range, and of a Scotch RG665 filter. A 600 line diffraction grating was mounted in place of a standard one. This equipment allows spectral identification of the singlet oxygen phosphorescence and measurement of the singlet-oxygen lifetime in the nanosecond and microsecond ranges. The filters employed are essential for eliminating from the infrared signal all harmonic contributions of the sensitizer emission in the 400–900 nm range. Solutions of photosensitizers were prepared with matched absorbances close to 0.2 at 355 nm. Following laser excitation, the singlet oxygen phosphorescence was followed at 1270 nm in the hundreds of nanoseconds and microseconds range. By extrapolating to time-zero the decays of the singlet oxygen emissions in ethanol, we obtain relative phosphorescence intensities at a given laser pulse energy. The measurements were repeated at various laser pulse energies and the intensities were correlated with the laser energy to ensure that a linear relation was observed (i.e., the experiments were made far from saturation of the signal). The relative intensities were converted to singlet oxygen quantum yields using the phosphorescence of singlet oxygen obtained after excitation of a reference: phenalenone in ethanol (Φ_Δ_ = 0.95 ± 0.02). The ratios of the slopes of the laser energy dependence for each photosensitizer versus phenalenone were used together with the singlet oxygen quantum yield to obtain the singlet oxygen quantum yields of the photosensitizers.

#### Photodegradation experiments

The photostability of porphyrins was investigated following the measurement of their optical absorption in aqueous solutions. Continuous irradiation of an aerated solution were carried out using the xenon lamp (XBO-150) through the 10 cm water filter and bandpass filter transmitting within >400 nm range, delivering 75 mW/cm^2^. The reaction progress was monitored by UV/Vis spectroscopy using a Hewlett Packard HP8453 spectrophotometer.

#### Detection of reactive oxygen species in solution

3’-*p*-(aminophenyl)fluorescein (APF) and 3’-*p*-(hydroxyphenyl)fluorescein (HPF) are selective probes for hydroxyl radicals. Singlet Oxygen Sensor Green® (SOSG) is a specific probe for singlet oxygen. Dihydroethidinum (DHE) is a probe for the identification of superoxide ion. These probes were employed for the detection of ROS after illumination of the PS. PS solutions were diluted to a final concentration of 10 μM per well. Next, each fluorescent probe was added to a well at a final concentration of 15 μM. PS solutions were irradiated with LED light for various time intervals. A microplate reader (Tecan Infinite M200 Reader) was used for acquisition of fluorescence signal immediately before and after illumination. When APF and HPF were employed, fluorescence emission at 515 nm was measured upon excitation at 490 nm. With SOSG, the corresponding values were 525 and 505 nm, and 480 nm and 580 nm for DHE, respectively.

### n-Octanol/PBS partition coefficients

The *n*-octanol/PBS partition coefficients were measured following shake-flask method with minor modifications. The photosensitizers were dissolved in *n*-octanol previously saturated with a solution of PBS. The same volume of PBS saturated with *n*-octanol was added and mixed on a vortex device and then the phases were separated by centrifugation. Next, the PBS/*n*-octanol phase was taken and diluted to obtain 0.5% of PBS/*n*-octanol content in the final solution. This solution was left into the ultrasonic bath. The fluorescence of each solution was measured using Fluorescence Spectrometer LS 55 (Perkin Elmer) and compared with calibration curve to obtain the concentration of the photosensitizer. Partition coefficients were calculated from the ratio c_oct_/c_PBS_, where c_oct_ and c_PBS_ are the concentrations of the porphyrin derivatives in the n-octanol and in the PBS.

### Binding to plasma proteins determined by fluorescence quenching

The binding of the PS to HSA was studied by spectrofluorometry at room temperature. An aqueous (phosphate buffer saline, PBS) solution of HSA was titrated with varying concentrations of the respective porphyrin solutions. HSA was excited at 295 nm and fluorescence was recorded between 300 nm and 400 nm. The systematic lowering of HSA fluorescence with increasing photosensitizer concentrations was noted and used in the determination of the binding constants and the number of binding sites on HSA according to Scatchard equation. The changes in HSA fluorescence intensity were related to porphyrin concentrations by the Stern-Volmer relationship. The binding constant (K_b_) of PS to lipoproteins was also determined using the spectroscopic titration method. The sets of steady-state emission spectra of PBS solutions with different concentrations of HDL and LDL, to which the porphyrin (5 μM) was added were measured. The fluorescence intensity of the dye increased upon its partitioning into the lipoproteins. To obtain K_b_, F versus [L] data were plotted and fitted to by a nonlinear regression routine.

### Characterization of PS-loaded polymeric micelles

Micellar formulations with Pluronic L121 were prepared according to a method recently described [40]. The micelles were characterized by Dynamic Light Scattering (DLS) using a Malvern Zetaziser Nano ZS system. The apparent diffusion coefficients of the micelles were obtained from the normalized time correlation function of the scattered electric field, g_(1)_(τ), using the cumulants analysis. An average value was obtained from repeated measurements for each sample (N = 3).

### Biological tests

The studies were performed on human lung adenocarcinoma (A549), murine colon carcinoma (CT26) and murine endothelial/vascular epithelium (2H11) cell lines. A549, CT26 and 2H11 cells were grown in full-strength DMEM with 4.5 g/L glucose, L-glutamine, sodium pyruvate and 3.7 g/L NaHCO_3_ (BioTech) with addition of 10% fetal bovine serum (BioTech, Poland) and supplemented by antibiotics (penicillin/streptomycin). Before the experiments, the cells were washed with PBS, removed by trypsinization, then re-plated into microplate (or culture flask) with appropriate cells density and maintained in a humidified atmosphere at 37 ^o^C and 5% CO_2_.

#### Photosensitizer cellular uptake determination

Cells were seeded on 96-plate microplate (10^4^ per well). After 24 h, the cells were incubated with different concentrations of photosensitizers for various time intervals from 2 h up to 24 h. The appropriate controls were included. The solutions of photosensitizers were prepared by diluting the porphyrin stock solution in PBS (hydrophilic compounds) or DMSO (amphiphilic derivative) or PS-Pluronic micellar formulation with the culture medium to the desired final concentration (5 μM). The highest concentration of DMSO in medium did not exceed 0.5%. After incubation, the cells were washed two times with warmed PBS and solubilized in 30 μl of Triton X-100 and 70 μl of DMSO/ethanol solution (1:3). The retention of cell-associated porphyrin was detected by fluorescence measurement with the microplate reader (Tecan Infinite M200 Reader).

#### Cytotoxicity in the dark

MTT cytotoxicity assay (which determines the mitochondrial activity), based upon the ability of living cells to reduce (3-(4,5-dimethylthiazol-2-yl)2,5-diphenyl tetrazolium bromide (MTT) into formazan was used to assess photosensitizer-mediated cytotoxicity. Two identical 96-well plates with A549, CT26 or 2H11 cell cultures seeded on 96-plate microplate (10^4^ per well) were used for each experiment. When the cells were attached to the plates, PS solution in growth medium (DMSO<0.5%) at concentrations between 0.1 to 100 μM was added to the culture. Treated cultures were incubated for optimal time (estimated experimentally) at 37°C in the dark. Next, the photosensitizer solution of each well was removed, cells were washed with PBS and 200 μl fresh full-strength culture medium supplemented with FBS and antibiotics were added to each well and cells were returned to the incubator for 24 h. MTT was dissolved at the concentration of 5 mg/ml in PBS. Briefly, MTT solution were added to each well (final concentration 0.5 mg/ml) and the microplates were further incubated for 3–4 h. Medium was then discarded and 100 μl of mixture of DMSO/methanol (1:1) was added to the cultures and mixed thoroughly to dissolve the dark blue crystals of formazan. Formazan quantification was performed using an automatic microplate reader (Tecan Infinite M200 Reader) by absorbance measurements with the 565 nm test wavelength. Each experiment was repeated three times. Data were expressed as mean absorbance value of six samples and standard error of the mean.

#### Photodynamic effect

Based on the cytotoxicity results, the nontoxic concentration of 20 μM was selected. Cells were seeded on 96-plate microplate (10^4^ per well). After 24 h cells were incubated in the dark with PS solution or PS-Pluronic micellar formulation in a culture medium for the time interval determined in cellular uptake study. After the incubation time, the cells were washed with PBS and irradiated with the 635±20 nm light emitting diode (Illuminator with adjustable light intensity, Instytut Fotonowy, Poland) for various time intervals. Next, the cells were washed with fresh medium and plates were returned to the incubator for 24 h. The phototoxicity was determined by a MTT assay (described above) performed 24 h after irradiation.

#### Intracellular detection of reactive oxygen species

APF and HPF were employed for intracellular detection of ROS formation during PDT experiments. Cells were seeded on 12-plate microplate (2·10^4^ per well). After being washed with fresh medium, cells were incubated in the dark with PS solution (20 μM) diluted in cell medium for appropriate incubation time. The cells were also incubated with APF/HPF for 2 h prior to the experiment. In all experiments, the fluorescent probes were present at the 25 μM concentration. Then, the cells were washed with PBS and irradiated with the red LED light. The microplate reader (Tecan Infinite M200 Reader) was used for acquisition of fluorescence signal before and immediately after illumination. The fluorescence emission at 515 nm was measured upon excitation at 490 nm.

#### Photodynamic inactivation of microorganisms

The microorganisms used in PDI were: *S*. *aureus* (8325–4), *E*. *faecalis* (ATCC29212), *E*. *coli* (K12), *P*. *aeruginosa* (ATCC19660), *S*. *marcescens* (ATCC14756) and *C*. *albicans* (DAY286). The planctonic bacteria cells were cultured in brain heart infusion broth (Sigma-Aldrich) and LB Broth/Lennox (BioShop Lab Science Products) in an orbital incubator (37°C, 130 rpm), until the optical density reached 0.5, which corresponds to approximately 10^7^ CFU per mL. The fungal yeasts were cultured in YPD Broth (BioShop) with overnight aeration at 30°C to reach *ca*. 10^6^ CFU/mL. For PDI experiments cell suspensions of bacteria or fungal yeasts were incubated with various concentrations of the porphyrins at pH 7.4 (PBS) for 1 h in the dark at room temperature. Aliquots (1 mL) were transferred to a 12-well plate and illuminated with a blue light. We used blue-LED array (420±20 nm) to deliver a light dose of 10 J/cm^2^ (measured with a power meter). Cells treated with photosensitizers in the dark were incubated covered with aluminum foil for the same time as the PDI groups (1 h). After illumination (or dark incubation) samples were shaken, diluted in PBS, mixed and plated (LB agar). Aliquots were taken from each well to determine the CFU value. Plates were streaked in triplicate and incubated for 12–36 h at 30 ^o^C (for fungal yeasts) or 37 ^o^ C (for bacteria) in the dark to allow colony formation. A control group of cells treated with light alone showed the same number of CFU as the absolute control (data not shown).

### Statistics

All values are expressed as average±SEM (standard error of the mean), which represents the standard deviation of the sample mean estimate of a population mean. All experiments were repeated at least three times with comparable results. The sample size in biological tests was N = 6–12 in each experimental group. Experiments were also repeated at least three times with comparable results. The t-test was used for the determination of statistical significance. Results were considered as statistically significant with a confidence level of 95% (p < 0.05). Statistical analysis was performed with the STATISTICA 12.5 (StatSoft Poland, Krakow).

## Results and discussion

### Photochemical, physicochemical and pharmacological characteristics

#### Photosensitizers

**Fig 1:**
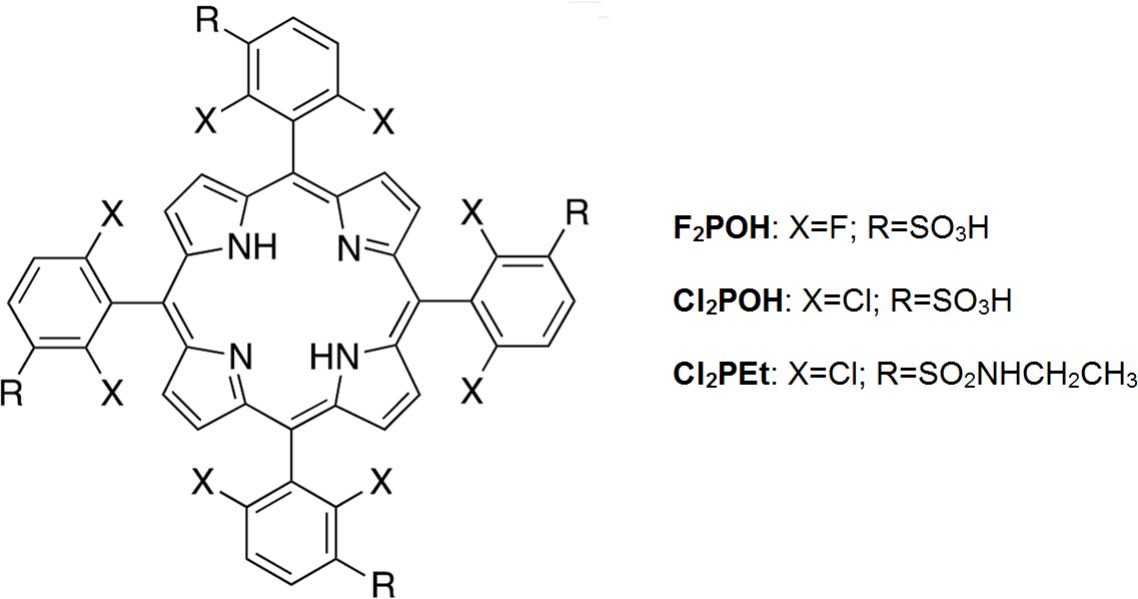
Chemical structures of investigated photosensitizers.

The synthesis of *meso*-substituted porphyrin derivatives 5,10,15,20-*tetrakis*(2,6-difluoro-3-sulfophenyl)porphyrin (**F**_**2**_**POH**), 5,10,15,20-*tetrakis*(2,6-dichloro-3-sulfophenyl)porphyrin (**Cl**_**2**_**POH**) and 5,10,15,20-*tetrakis*(2,6-dichloro-3-*N*-ethylsulfamoylphenyl)porphyrin (**Cl**_**2**_**PEt**) have been described before [41, 42]. They were prepared according to previously described routes, *via* nitrobenzene [43, 44] or nitrobenzene modified NaY methods [45], followed by chlorosulfonation and nucleophilic attack of water or ethylamine. Their structures are presented in Fig 1.

The substituents in the phenyl rings modulate photophysical properties and lipophilicity that then strongly influence their biological activity. The halogen atoms in the *ortho* positions enhance intersystem crossing to the triplet state and increase the triplet quantum yield (Ф_T_). Additionally, the steric interaction between the halogen atoms and hydrogen atoms in β positions, increases the angle between the macrocycle and the phenyl ring, and diminishes the tendency of porphyrin derivatives to aggregate [4, 46]. Besides the electronic effects derived from halogen substituents, the peripheral, polarity-tunable groups (sulfonic acid *vs*. sulfonamide) can alter the biological properties as required for a wide variety of biomedical applications, *e*.*g*. PDT and PDI.

#### Photophysical and photochemical properties of halogenated porphyrins

**Fig 2:**
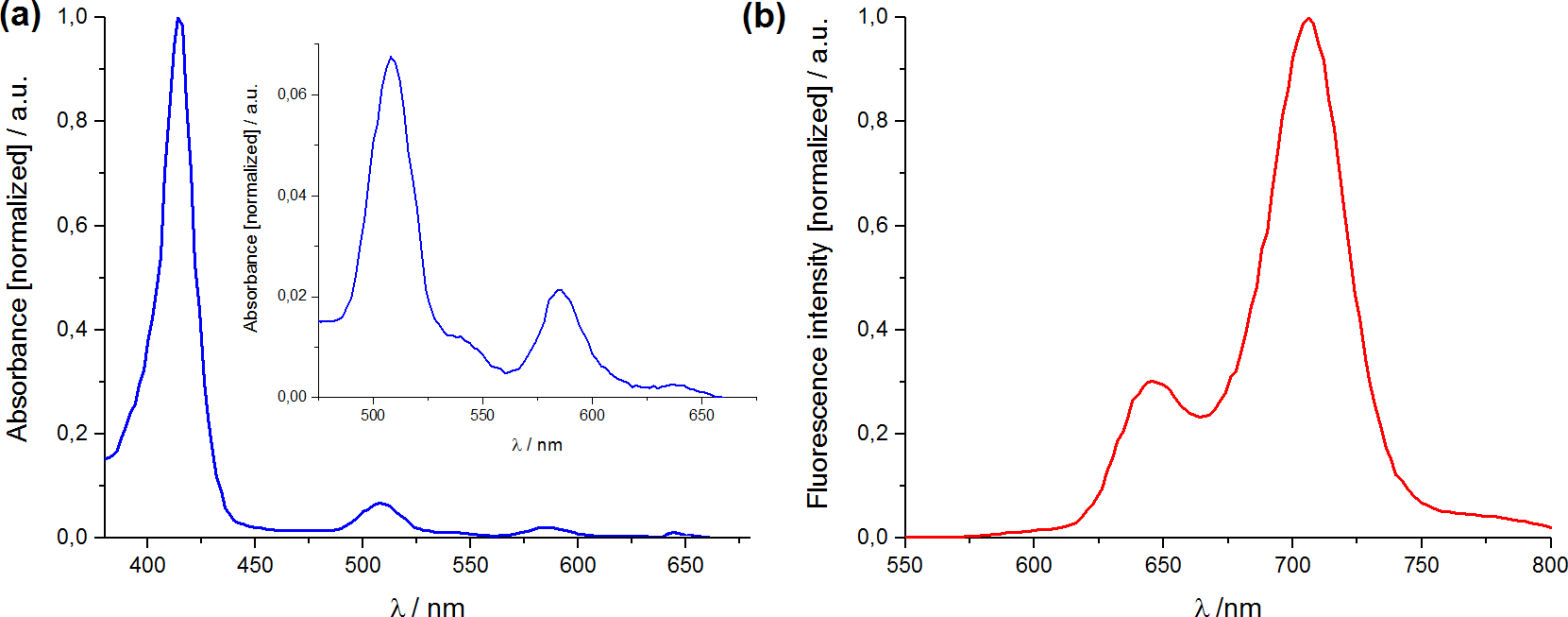
**Normalized electronic absorption (a) and fluorescence (b) spectra of fluorinated sulfonated porphyrin (F**_**2**_**POH) registered in EtOH at room temperature.** Inset in 2b shows the absorbance profile with multiplied values at λ>450 nm.

The absorption spectrum of each compound is similar and shows five bands characteristic for this class of porphyrins (according to Gouterman model): an intense Soret band around 420 nm and four other less intense Q-bands at lower energies [4]. Fig 2 shows representative absorption and fluorescence spectra of F_2_POH in ethanol.

The molar absorption coefficients were determined from Beer’s law and exceed 10^5^ M^-1^cm^-1^ near 420 nm. The porphyrins have the characteristic spectra of the *phyllo* type—the Q-bands intensities follow the sequence ε_I_ >ε_II_ <ε_III_ > ε_IV._ The absorption and fluorescence properties of all the studied halogenated porphyrins are listed in Table 1.

**Table 1:** Photophysical properties of F_2_POH, Cl_2_POH and Cl_2_PEt in EtOH.

	Absorption ε /M^-1^∙cm^-1^; λ_max_ /nm;					Fluorescence		Φ_Δ_
	B(0,0)	Q_y_ (0,1)	Q_y_ (0,0)	Q_x_ (0,1)	Q_x_(0,0)	λ_max_ /nm	Φ_F_	
**F**_**2**_**POH**	3.29∙10^5^ (420)	7.44∙10^3^ (508)	1.48∙10^3^ (532)	2,29∙10^3^ (584)	1.00∙10^3^ (638)	646, 706	0.040	0.71
**Cl**_**2**_**POH**	1.16∙10^5^ (420)	7.16∙10^3^ (514)	1.64∙10^3^ (546)	2,13∙10^3^ (592)	5.07∙10^2^ (646)	650, 716	0.015	0.98
**Cl**_**2**_**PEt**	6.24∙10^5^ (418)	6.20∙10^4^ (514)	1.17∙10^3^ (546)	2,40∙10^3^ (592)	1.53∙10^3^ (646)	652, 710	0.017	0.85

All photosensitizers display some absorption maxima within the phototherapeutic window (ε≈10^3^ M^-1^cm^-1^ at 638–646 nm). The longest-wavelength absorption band is essential for anticancer approach, because red light has sufficient tissue penetration ability. On the other hand, the intense absorption at 420 nm can be useful for antimicrobial evaluation. Light at 630 nm has a higher optical penetration depth than light at 420 nm (1.7 mm vs. 0.4 mm in skin). It is possible to calculate that, for the same incident light fluence at the skin surface, the light intensity 2 mm beneath the skin is ca. 50 times larger at 630 nm than at 420 nm. However, the molar absorptiom coefficients of porphyrins are typically 200–400 times higher at 420 nm than at 630 nm. The results of these two factors is that, at a depth of 2 mm beneath the skin, the number of photons absorbed by a porphyrin photosensitizer at 420 nm is ca. 5 times higher than the number of 630 nm photons. Deeper in the skin the situation is inversed. However, this explains why the treatment of actinic keratosis (a lesion located less than 2 mm deeper in the epidermis) is as effective with Levulan® Kerastick® using BLU-U® ate 417 nm, as it is with Metvix® using Aktilite at 630 nm. It is unlikely that in the clinic PDI of microorganisms will be used to treat infections more than 4.5 mm in depth. Hence, it is possible that 420 nm light is at least as effective as 630 nm light to treat such infections when porphyrin photosensitizers are used. When dealing with decontamination of invasive medical instruments, it is even more likely that 420 nm light will be more effective, for porphyrin photosensitizers, than 630 nm light.

The fluorescence spectrum in Fig 2B is typical for halogenated tetraphenylporphyrins showing a small Stokes shift (~4–8 nm) in ethanol. Table 1 also presents the fluorescence emission maxima and fluorescence quantum yields determined for the PSs studied in this work. The fluorescence quantum yields increase in the order Cl_2_POH ≈ Cl_2_PEt < F_2_POH, revealing that the presence of heavier atoms in the *ortho* position of the phenyl ring in the porphyrin macrocycle increases the rate of intersystem crossing to the triplet state and reduces the fluorescence quantum yield [16, 28, 47]. Chlorine atoms possesses significantly higher spin-orbital coupling constant than fluorine atoms making ISC more favorable for chlorinated compounds. The fluorescence quantum yields determined for halogenated porphyrins are significantly lower than that of non-halogenated reference TPP, Φ_F_ = 0.10 [46].

#### Reactive oxygen species generation

**Fig 3:**
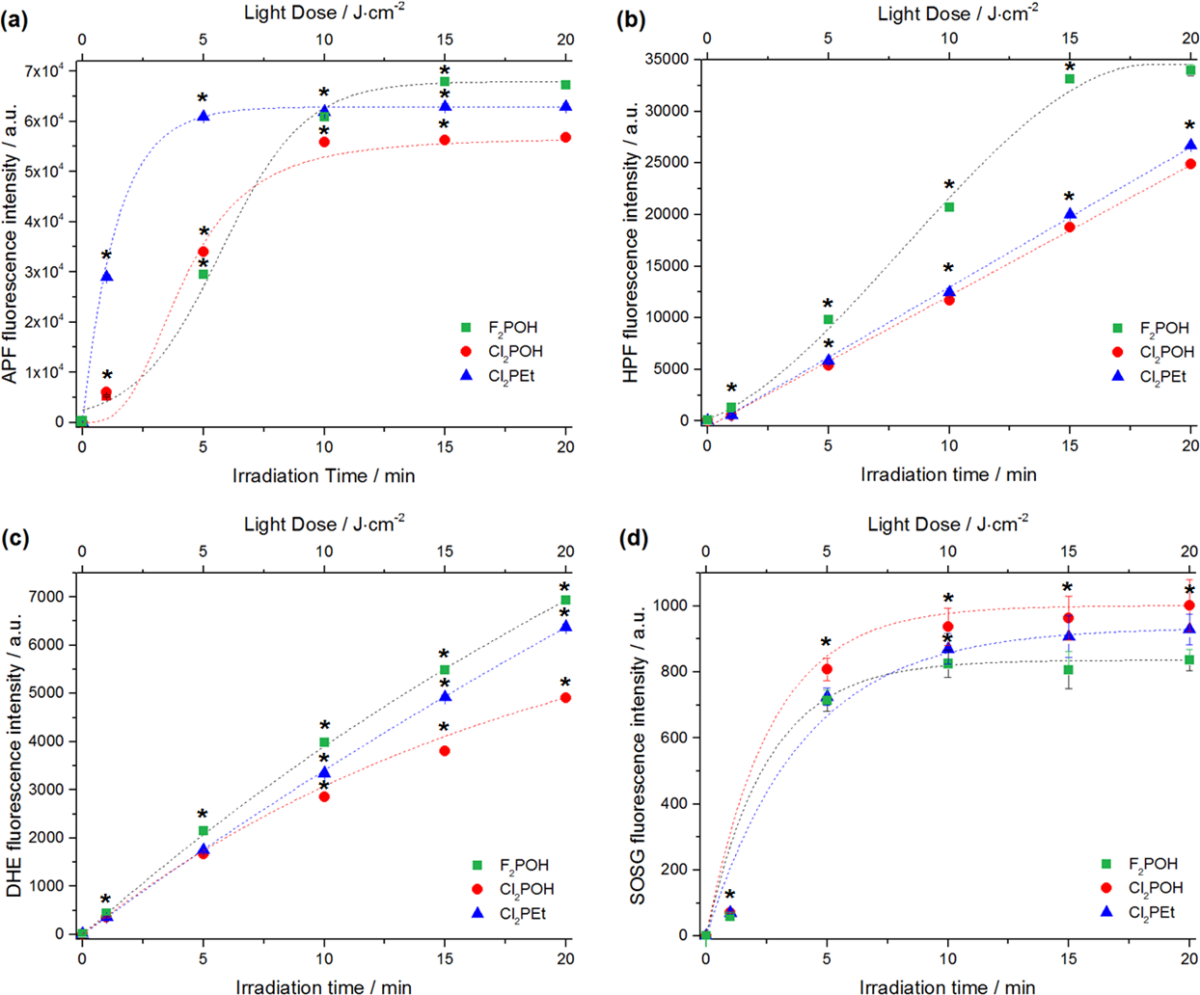
Reactive oxygen species generation. Fluorescence generated from selected ROS probes (10 μM): (a) APF, (b) HPF, (c) DHE, (d) SOSG during irradiation of photosensitizer solution (5 μM, EtOH:PBS *ca*. 1:99 *v/v*). Presented data are expressed as mean value (N = 6) ±SEM. The label (*) represents statistically significant difference (P<0.05). The t-test has been used for the statistical significance determination.

Considering the potential applications of these porphyrins, their ability to generate ROS *via* type I (hydrogen or electron transfer) or type II (energy transfer) photoreactions was evaluated. The singlet oxygen quantum yields (Φ_Δ_) were first determined directly by measuring the singlet oxygen phosphorescence decay at 1270 nm. As shown in Table 1, the singlet oxygen quantum yield of the fluorinated PS (Φ_Δ_ = 0.71) is significantly lower than those observed for the dichloro-substituted PS (Φ_Δ_ = 0.98 for Cl_2_POH, Φ_Δ_ = 0.85 for Cl_2_PEt, respectively). This is consistent with the spin-orbit coupling constants of the atoms in the *ortho* positions: ζ = 0.24 for H, ζ = 269 for F and ζ = 586 for Cl [25]. Hence, the internal heavy atom effect accelerates the S_1_→T_1_ intersystem-crossing rate and increases the triplet quantum yields (Ф_T_) of investigated photosensitizers, which are maximized with chlorine atoms in the *ortho* positions. Even though ^1^O_2_ (^1^Δ_g_) is thought to be the dominant cytotoxic ROS in porphyrin-mediated PDT, other type of ROS may be also generated by electron or hydrogen atom transfer. We recently reported that fluorinated sulfonamide porphyrin derivatives generate hydroxyl radicals in solution as well as in a cellular environment [24, 26]. This motivated the study of ^1^O_2_, hydroxyl radical and superoxide ion generation by our PS using SOSG, APF, HPF and DHE fluorescent probes (Fig 3). These fluorescent probes indicate the relative contribution of the different ROS to the photodynamic effect of the series of PS. While SOSG is mainly sensitive to ^1^O_2_, the APF and HPF are more sensitive to hydroxyl radicals, but APF is also sensitive to other ROS including ^1^O_2_ [48]. Dihydroethidium (DHE) is a redox-sensitive probe used to detect (intracellular) superoxide anion [49]. Fig 3 shows the fluorogenic response of each probe after activation by the porphyrins. The fluorescence of APF, HPF and DHE suggests that all porphyrins produce radical species through type I photoreaction in addition to singlet oxygen. The intensity of SOSG follows that of singlet oxygen phosphorescence: Cl_2_POH produces more singlet oxygen and F_2_POH produces less. The role played by of each one of radical species (superoxide ion, hydroxyl radicals) and singlet oxygen in the oxidative stress induced during photodynamic action also depends on the presence of reductants or antioxidant enzymes, such as NADH, catalase or superoxide dismutase (SOD) [50]. Thus, depending on experimental conditions, both ^1^O_2_ and oxygen-centered radicals may be involved in the photodynamic activity and cause oxidative damages to the targeted cells. Hence, both type I and type II mechanisms may occur competitively and their relative contributions will depend on the photosensitizer, substrates and environment.

#### Photobleaching

In order to establish the rate of photodegradation of our porphyrins, photostability studies were performed at the same irradiation conditions as used in the biological studies. The UV-Vis spectra showed that the absorbance intensities of the Soret bands did not significantly changed during irradiation (S1 Fig). Under these conditions, all compounds showed a high photostability. Other widely used porphyrin derivatives, such as HPD and PpIX are more prone to photooxidation when irradiated under the same conditions.

#### Lipophilicity

Partition coefficients were obtained experimentally by the modified shake-flask method and are summarized in Table 2 in terms of their logarithmic values (logP). These results indicate that the sulfonic acid substituents significantly enhance hydrophilicity.

**Table 2:** Partition coefficients, fluorescence quenching data for the interaction of the porphyrins with HSA and lipoproteins (HDL, LDL) with binding constants (K_b_).

	Lipophilicity	PS-Plasma Protein Interaction				
		HSA			Lipoproteins	
	logP	*K*_SV_/ 10^5^M^-1^	*K*_b_/ M^-1^	*n*	*K*_b_ HDL / (mg/mL)^-1^	*K*_b_ LDL / (mg/mL)^-1^
**F**_**2**_**POH**	-1.74	2.24	0.58	1	14.81	8.39
**Cl**_**2**_**POH**	-1.80	4.66	0.74	1	12.84	8.88
**Cl**_**2**_**PEt**	1.84^a^	6.62	0.82	1	28.38	19.93

^[a]^ See also Ref. [42].

#### Investigation of PS-plasma protein interactions

**Fig 4:**
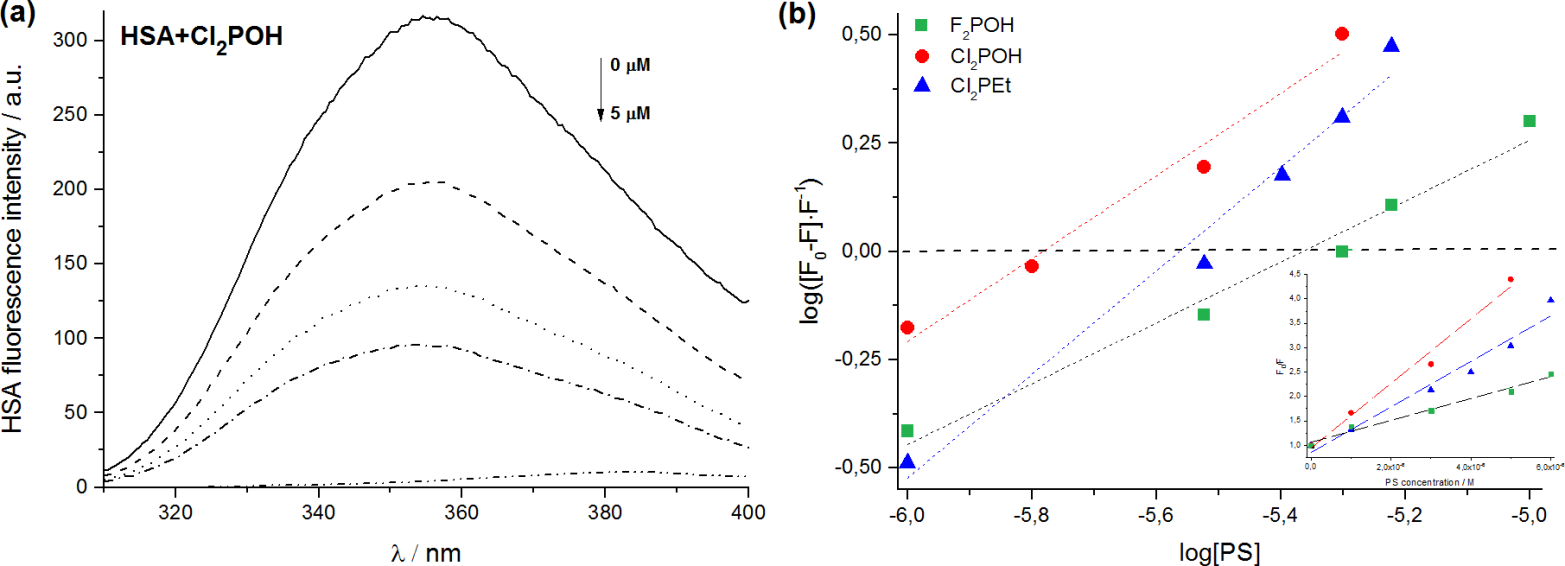
(a) Fluorescence spectra of HSA in the presence of various PS concentrations, (b) Scatchard analysis performed for PS-HSA interaction and Stern-Volmer plots (inset).

The binding of the photosensitizers to human serum albumin (HAS) plays an important role in their biodistribution and PDT efficacy *in vivo*. Many compounds, especially amphiphilic drugs, have high affinity to HSA and can form stable complexes with HSA [51]. Porphyrins are usually introduced in the blood from relatively high concentration solutions, which may diminish their action or even cause adverse effects. Interactions with plasma proteins may control the efficacy and biodistribution of porphyrins [52]. In addition to serum albumin, which is the predominant protein in plasma, it is widely recognized that the very-low density (VLDL), low-density (LDL) or high-density lipoproteins (HDL) may also play important roles in the interactions with photosensitizers in the serum. For instance, the binding with lipoproteins may increase the selectivity of drugs towards tumor tissue, due to enhanced expression of specific LDL receptors in many types of neoplastic cells. In addition, it has been reported that the efficiency and tumor targeting by photosensitizers may be improved by increasing the hydrophobicity of the molecule. In particular, hydrophobic photosensitizers mainly bind to LDL and can be successfully incorporated into the apolar core of LDL. Kessel et al. performed the comprehensive studies on plasma binding properties of sulfonated derivatives of tetraphenylporphyrin and indicates that PS with one or two (adjacent) sulfonates bound to VLDL, LDL and HDL components of plasma, while the tri- and tetra-sulfonated analogs bound progressively more to albumin [52]. Therefore, the interaction of PS with plasma proteins, and especially with HSA, is of high importance to establish safe and effective dosages. The binding of our porphyrins to HSA and lipoproteins was followed by steady-state fluorescence measurements (Fig 4, S2 Fig) Fluorescence quenching experiments were used to assess the association of the porphyrin with the HSA binding sites and form PS-HSA complexes.

The fluorescence spectra were analyzed to calculate Stern–Volmer constants (*K*_SV_) for all porphyrins. Following Eq (1) in Materials and Methods, *K*_SV_ was obtained from the slope of the plot (3b, inset). Moreover, the binding constant (*K*_b_) and the number of binding sites (*n*) were estimated, Table 2. The binding of the porphyrins to HSA is stronger for the less polar porphyrin (Cl_2_PEt) than for the hydrophilic porphyrins (Cl_2_POH, F_2_POH). An hydrophobic region seems to stabilize PS-HSA complexes and is opposed by hydrophilic groups that increase aqueous solubility [52]. The results also suggest that the more lipophilic sulfonamide derivative indicates the stronger interaction with lipoproteins (LDL, HDL). The differences in plasma proteins binding behavior affect the localization of photosensitizers in cells. The transport and distribution of PS may be supported *via* two mechanisms, which one is mediated by lipoprotein binding and leading to porphyrin accumulation in intracellular compartments and another associated with albumin binding may results to porphyrin accumulation in membrane sites.

### Biological studies

#### Micellar formulation of hydrophilic photosensitizers

Previous studies carried out in our research group showed that Pluronic copolymers (P123 and F127) provide interesting drug delivery vehicles for photosensitizers not soluble in water [34]. On the other hand, it was shown that a micellar formulation containing the hydrophobic copolymer Pluronic L121 improved the delivery to cancer cells of a hydrophilic fluorinated phthalocyanine derivative. This seemed to be a more appropriate approach to the PS described in this work, and Pluronic-based formulations were prepared as reported in the literature [40]. Addition of L121 to the porphyrin solution revealed a dispersion of the oil phase in the phosphate buffer which is typical for Pluronics with long propylene oxide (PO) chains and short ethylene oxide (EO) chains [53]. Due to the strong interaction of Pluronic L121 copolymer with lipid molecules, the presence of complex film at the oil-water interfaces increase the adsorption and entry of the dispersed drug molecules across the cell membrane, facilitating its transport and improving the intracellular uptake [54]. We characterized the Pluronic L121-based formulation using dynamic light scattering (Table 3, S3 Fig) and found that for F_2_POH particle distribution is homogeneous with average hydrodynamic diameter *ca*. 115 nm. Cl_2_POH-L121 indicate different densities of the heterogeneous particles and reveal formation of micellar cluster and large aggregates (>1000 nm) usually observed for non-monodisperse systems such as these polymeric nanoparticles.

**Table 3:** Properties of the PS-Pluronic-based formulation: Hydrodynamic diameter and zeta potentials of prepared micelles.

	Particle Diameter / nm	Zeta Potential ζ / mV
**F**_**2**_**POH-L121**	115.4±20.7	-2.24±0.1
**Cl**_**2**_**POH-L121**	92.8±0.2 (10%); 1320±124 (90%)	-1.51±0.3

It is well known that several parameters (such as the block-length ratio of the hydrophilic to the hydrophobic block, hydrophobicity of the apolar block, molecular weight etc.) influence the type and size of the nanostructure formed upon self-assembly. However, short hydrophilic blocks can result in the formation of large structures upon hierarchical aggregation of smaller micelles [55].

#### The impact of polymer addition on ROS generation

**Fig 5:**
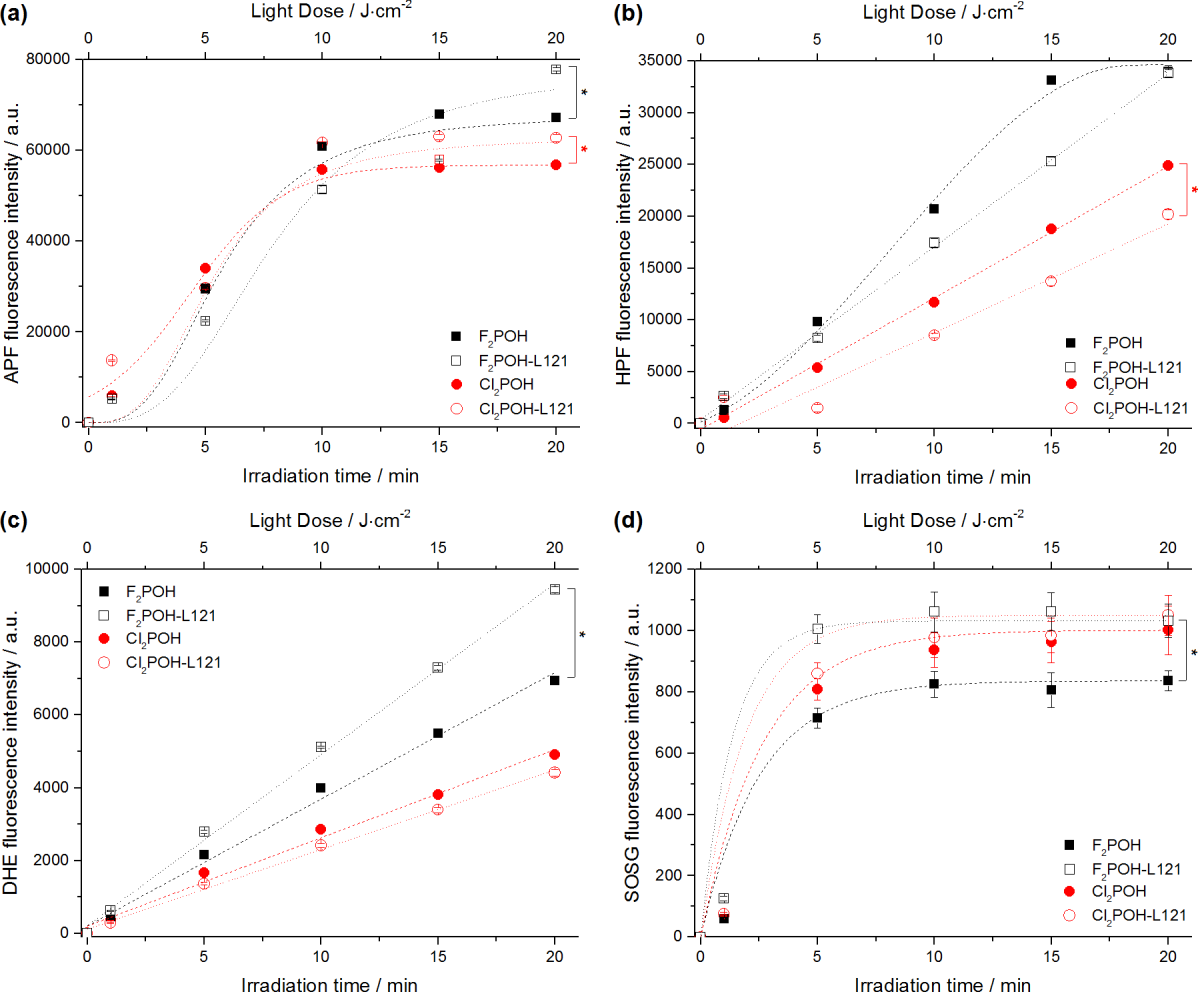
**Fluorescence generated from ROS probes (10 μM): (a) APF, (b) HPF, (c) DHE, (d) SOSG during irradiation of porphyrin solution and porphyrin-L121 formulation at concentration at 5 μM.** The data are expressed as mean value (N = 6) ±SEM. The label (*) represents statistically significant difference (p<0.05).

The influence of Pluronic L121 on reactive oxygen species generated by hydrophilic porphyrins was evaluated and is illustrated in Fig 5. The changes in ROS level during irradiation were determined in order to evaluate the ability of the micelles to improve PDT efficacy of hydrophilic halogenated porphyrins. The fluorescence intensity of SOSG, APF, HPF and DHE generally increased with increased light dose for L121-formulated photosensitizer, but that effect was more evident for F_2_POH.

#### Time-dependent cellular uptake

**Fig 6:**
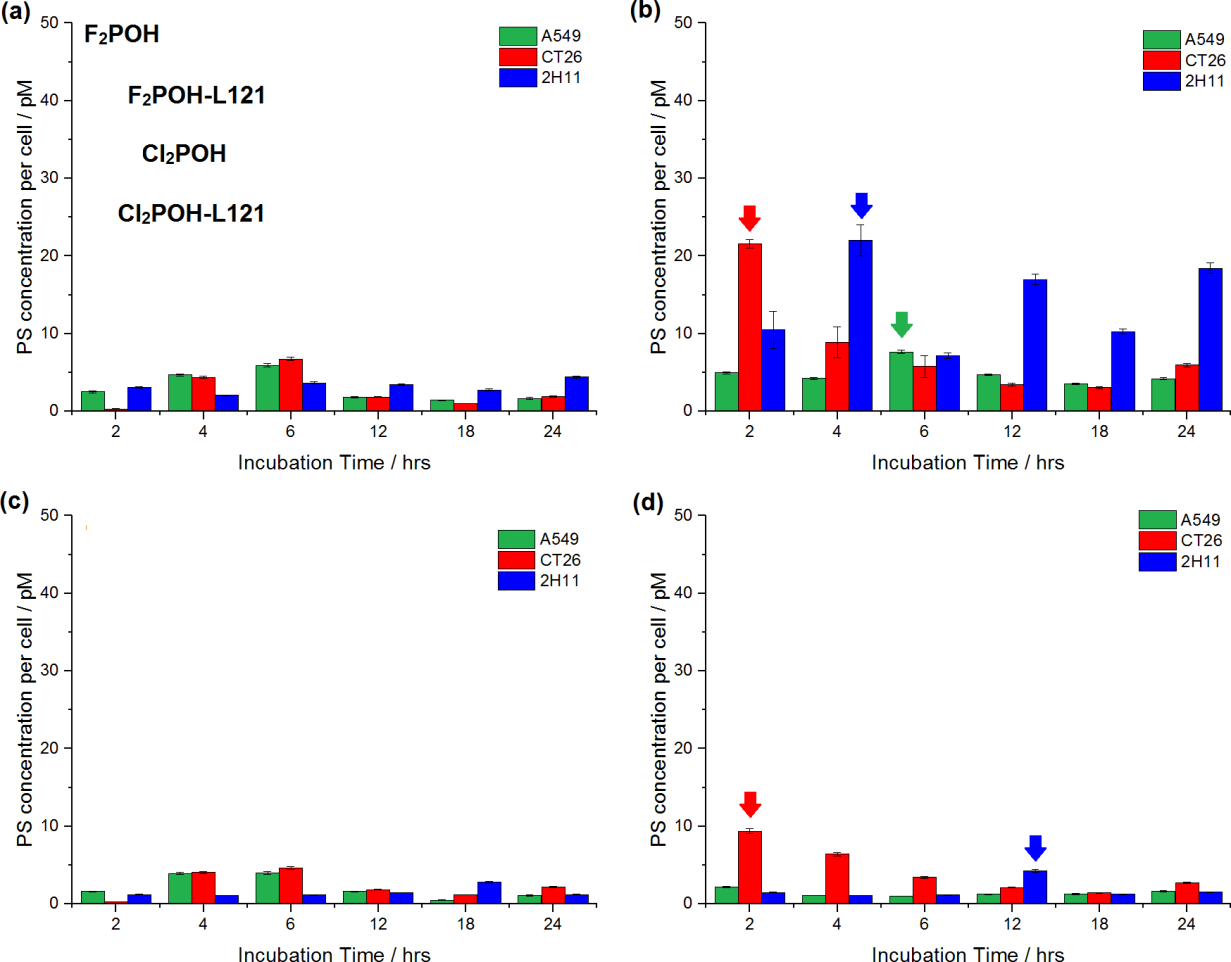
**Time-dependent uptake of hydrophilic photosensitizers (F**_**2**_**POH, Cl**_**2**_**POH) without (a, c) and with (b, d) various micellar formulations by A549, CT26 and 2H11 cells.** The data are expressed as mean value (N = 12) ±SEM.

Hydrophilic photosensitizers have more difficulties in crossing biological membranes and their intracellular accumulation may be lower. On the other hand, they are generally less prone to self-aggregation in the plasma than hydrophobic ones. Taking into account the fact that cellular uptake of porphyrin is limited by their water solubility we decided to evaluate the impact of the micellar formulation on the accumulation of PS in cells. The cellular distribution of Pluronics is strongly affected by hydrophobicity, concentration, cell type and incubation time. For instance, Pluronic P123 is retained in the plasma membrane and slowly transported into the cells, resulting in endocytic localization. Fig 6 indicates that the L121 micellar formulation leads to an increased cellular uptake of hydrophilic photosensitizers in cancer cells. Moreover, we observe a reduced optimal accumulation time for CT26 (2 h for micellar mixture *vs*. 6 h for free PS) and 2H11 (from 6 h to 4 h in the case of F_2_POH and from 18 h to 12 h for Cl_2_POH).

**Fig 7:**
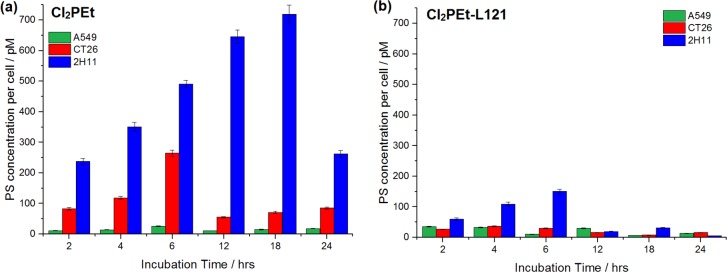
**Time-dependent cellular uptake of free Cl**_**2**_**PEt (a) and after micellar (L121) modification (b) in A549, CT26 and 2H11 cells.** The data expressed as mean value (N = 12) ±SEM.

The highest intracellular accumulation of sulfonamide derivative (Cl_2_PEt) was reached without polymer addition (Fig 7). The sulfonamide groups in the structure of the macrocycle suffice to promote cellular accumulation. The uptake of this PS in micelles stabilizes after 4–6 h of incubation, while the accumulation from serum-DMSO medium continues to increase for 20 h of incubation. This higher uptake for longer incubation times may be biased by the aggregation of the photosensitizer followed by disaggregation when the cells are treated before the fluorescence measurements.

#### Dark cytotoxicity of photosensitizers

The dark cytotoxicity of the photosensitizers was evaluated using MTT assay. Various concentrations of the porphyrin (0–100 μM) were added to cell cultures and the cells were incubated in the dark for 24 h. The analysis of cellular response to increased concentration of photosensitizers showed no significant dark cytotoxicity for three photoactive drugs in this concentration range (S4 Fig). 100 μM of F_2_POH induced only 25% of cytotoxicity of A549, CT26 and 2H11 cells, thus in all subsequent PDT experiments used the sub-lethal concentration of 20 μM to induce cell photodamage.

#### Photodynamic activity against cancer cells

**Fig 8:**
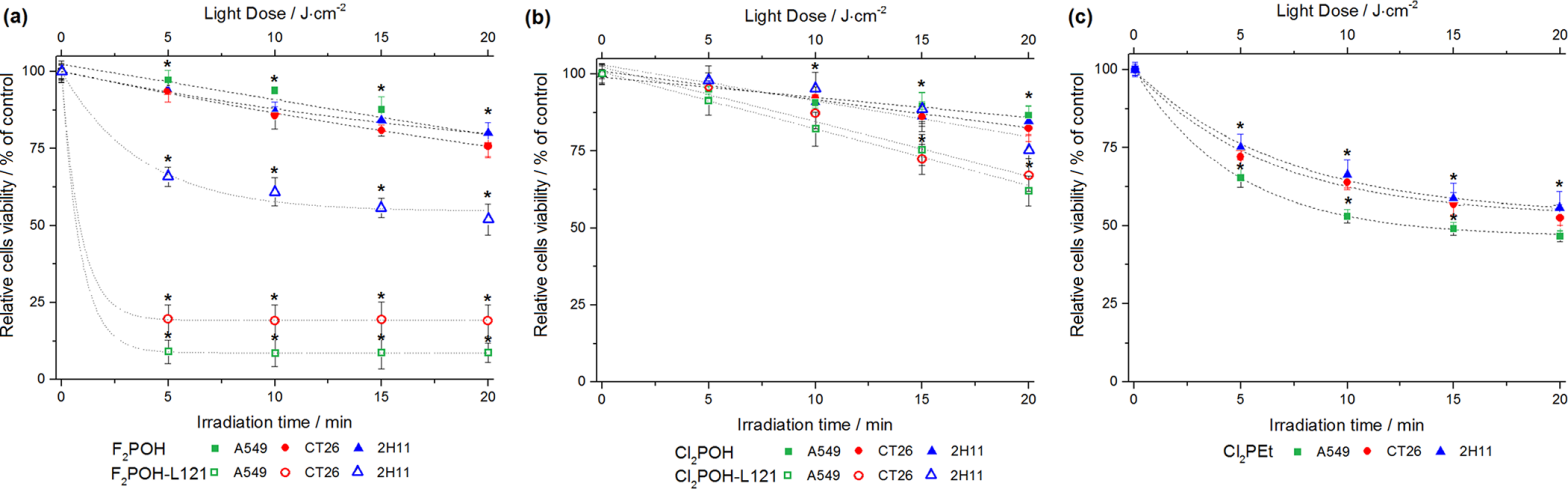
Photodynamic effect induced by studied photosensitizers in the absence (or in the presence) of poloxamer addition against A549, CT26 and 2H11 cells. The data are expressed as mean value (N = 12) ±SEM. The label (*) represents statistically significant difference (p<0.05).

Cells incubated with non-cytotoxic concentrations of photosensitizer for the time interval selected on the basis of cellular uptake were subject to different light doses to evaluate phototoxicity. Fig 8 shows that photodynamic effect depends on the type of cells, type of PS and light dose. Moreover, Pluronic L121 micelles increased the efficacy of the hydrophilic photosensitizers without additional cytotoxicity. This is especially evident for F_2_POH. A549 cells were in general more sensitive to PDT. 20 μM of F_2_POH-Pluronic L122 with light doses 10–20 J/cm^2^ lead to *ca*. 90% mortality of A549 and CT26 and 50% in case of murine endothelial cells. These results show that the Pluronic-L121 is an advantageous drug delivery system for PDT with hydrophilic photosensitizers.

**Fig 9:**
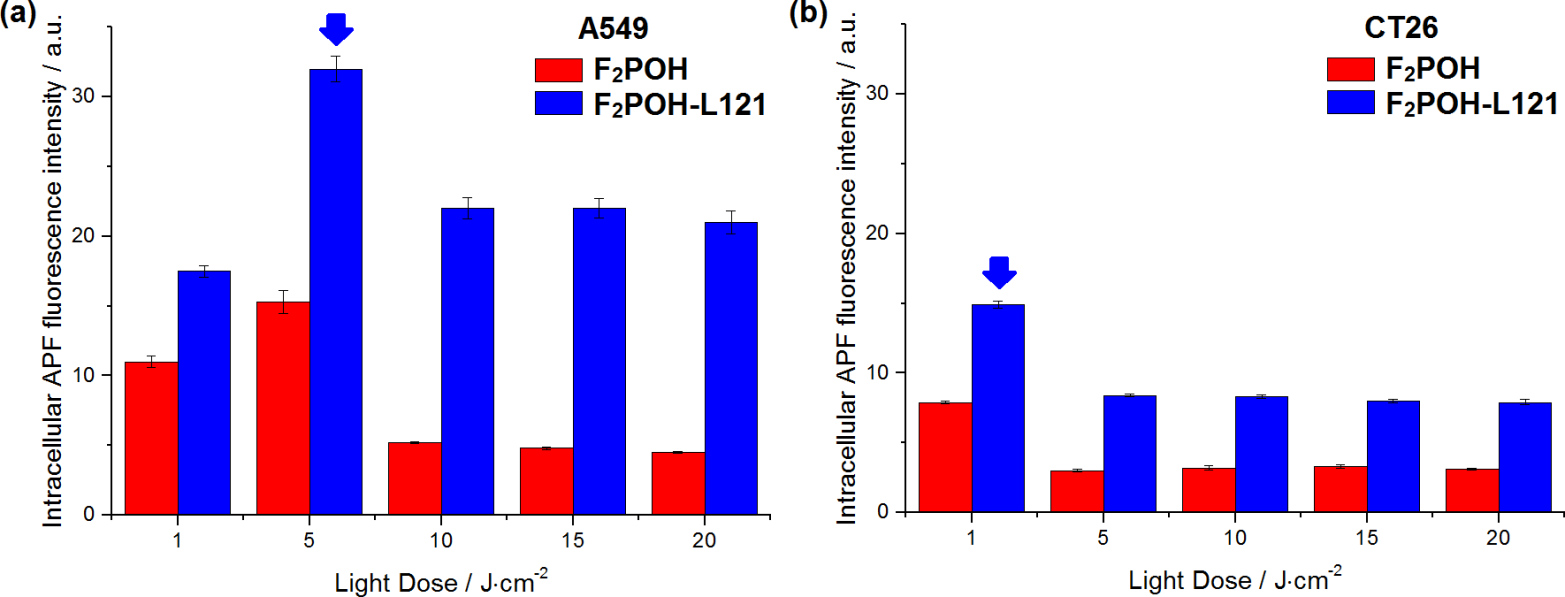
**Evaluation of ROS generation after photodynamic effect mediated by F_2_POH in two different formulations (PBS *vs*. Pluronic L121 micelles) under comparable experimental conditions: fluorescence generated from APF (25 μM) in (a) A549 and (b) CT26 cells incubated PS for optimal (comparative) incubation time and irradiated with various red light doses.** The data expressed as mean value (N = 6) ±SEM.

The relatively low photodynamic efficacy of F_2_POH and Cl_2_POH in aqueous media is related with the hydrophilicity of these compounds, that reduces their ability to penetrate cell membranes. This limitation is not experienced by Cl_2_PEt, which has a much higher cellular uptake. Interestingly, once the problem of cellular uptake of F_2_POH is solved by the formulation with poloxamer Pluronic L121, its phototoxicity becomes at least as high as that of Cl_2_PEt but at a much lower concentration per cell. However, phototoxicity is not simply related with the concentration of photosensitizer per cell. Fig 6B shows that the highest concentration of F_2_POH in the F_2_POH-Pluronic formulation is observed for 2H11 cells and the lowest was obtained for A549 cells, but the latter cells are more sensitive to PDT. Fig 9 shows the level of oxidative stress induced by ROS as determined *in vitro* with APF and HPF fluorescent probes, which gives a clue to this puzzle: the higher concentration of the PS is not necessarily related to the highest oxidative stress. As seen from APF and HPF probes, the ROS generation in A549 cells is higher than in CT26 cells, although more PS molecules were internalized by CT26 cells. A549 cells (characterized by low SOD activity) are especially sensitive to photosensitizers that generate superoxide ions, and their phototoxicity can be potentiated by the inhibition of SOD [50]. On the other hand, CT26 cells have higher SOD activity and can upregulate catalase, thus conferring higher resistance against Type I processes. Although the ultimate phototoxicity of the PS will depend on the cell environment, it is nevertheless clear that the formulation of hydrophilic photosensitizers with Pluronic L121 increases their phototoxicity.

#### Photoinactivation of microorganisms

**Fig 10:**
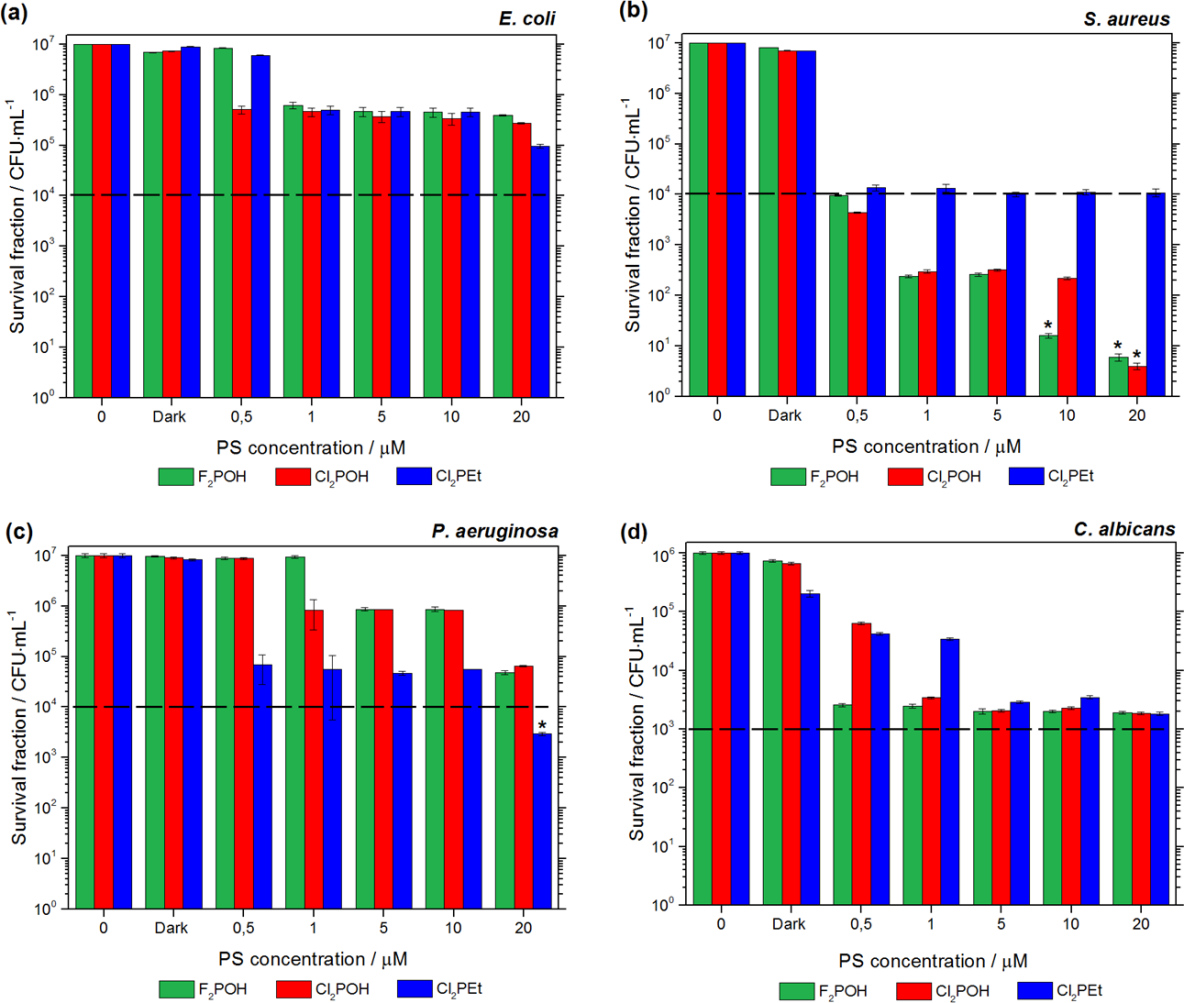
PDI of microorganisms mediated by halogenated porphyrin derivatives. Cells were incubated with PS for 1 h and exposed (or not) to 10 J/cm^2^ of visible light (420±20 nm). The dashed black line shows viability values for 99.9% (3 logs) inactivation of microorganisms.

Photodynamic inactivation of pathogenic microorganisms including Gram-positive, Gram-negative bacteria and fungal yeast was conducted in PBS cell suspensions incubated with different photosensitizer concentrations. The best way to compare the phototoxicity of a group of photosensitizers with very different potencies is to apply a wide concentration range and determine the cell survival with a single light dose (PDI) or without light (dark toxicity). The results for the selected microbial species with the three porphyrins are shown in Fig 10. In most cases, no toxicity was found after 1 h of incubation in the dark with the concentrations of photosensitizers employed in this study. The exception is *C*. *albicans* which showed an approximately 1 log reduction when incubated with Cl_2_PEt. The sulfonamide moieties of this halogenated porphyrin may act as competitive inhibitor of the dihydropterate synthase (DHPS) involved in folate synthesis. Due to its intrinsic sulfonamide properties, Cl_2_PEt has some by antifungal activity in the dark. The viability of microbial cells was not affected by irradiation without photosensitizer (data not shown). The photodynamically-induced death of prevalent pathogens was strongly dependent on the PS concentration and delivered light dose/illumination time. *S*. *aureus* (Fig 10B) was the most susceptible strain, with a 6 logs reduction in colony forming units (CFU) after 10 min irradiation with 20 μM of F_2_POH. Similar results were obtained with Cl_2_POH. The superior activity of these compounds toward Gram-positive *S*. *aureus* can be related to their negatively charged sulfonic groups. In contrast, Gram-negative species as well as fungal yeast stem to be less susceptible to PDI, but performed research allow as to determine the experimental conditions necessary for 99% (2 logs) inactivation. *E*. *coli* cells were the most difficult to eradicate (Fig 10A). A concentration of 20 μM Cl_2_PEt gave a reduction of 99% (2 logs) in the survival after 10 min irradiation. This PS was most effective against *P*. *aeruginosa* (up to 3 logs in bacteria CFU killing). The photodynamic activities of F_2_POH and Cl_2_POH towards *E*. *coli* and *P*. *aeruginosa* were very similar and resulted in 1.5–2 logs decrease in survival. The antimicrobial activity was also investigated against *E*. *faecalis* and *S*. *marcescens* (S5 Fig). Both sulfonic acid derivatives, at the lowest concentration (0.5–1 μM) have shown relevant decrease of *C*. *albicans* survival (~3 logs) after 10 min irradiation.

Relatively low PDI efficacy against Gram-negative bacteria has been reported for other PSs [3, 56]. It is related to the difference in porosity and composition as cell wall structure in Gram-positive and Gram-negative bacteria. The thicker and relatively porous cell wall of Gram- positive bacteria is made of interconnected peptidoglycan which is located immediately outside of cytoplasmic membrane. It is responsible for the rigidity of the cell wall but it is not considered to be a limiting permeability barrier for small molecules. ROS produced by illumination in cell environment can cross easily into the cytoplasm. In contrast, Gram-negative bacteria have an outer-membrane that contain lipopolysaccharide which lowers the membrane permeability for lipophilic compounds. Furthermore, the negatively charged lipopolysaccharide surface acts as static barrier for neutral and anionic molecules. The moderate PDI effect towards *C*. *albicans* may be due to the fact that fungal cell walls have a relatively thick layer of β-glucan which decreases PS diffusion into cytoplasm. We also tested the activity of encapsulated PS with Pluronic L121, but no significant differences were observed in comparison to studies without poloxamer addition (S6 Fig). We infer that the Pluronic L121-formulated PS was unable to drive porphyrin through the cell outer-membrane of the bacteria. Moreover, the molecular size and properties of the polymer and micellar formulation may hinder antimicrobial efficacy, as reported for phthalocyanine-based photosensitizer AIPcCl incorporated in Pluronic P123 [57].

## Conclusions

A set of tetraphenylporphyrin (TPP) derivatives was selected and characterized for PDT and PDI. The incorporation of halogen (fluorine or chlorine) atoms in the phenyl rings of TPP increases the probability of intersystem crossing and the introduction of sulfonic or sulfonamide groups modulates their water-solubility and the interaction with the biological membranes. Furthermore, these substituents also prevent the aggregation and increase photostability of photosensitizers. F_2_POH, Cl_2_POH and Cl_2_PEt combine these chemical and photochemical properties with a low toxicity in the dark over the micromolar concentration range and a tendency to accumulate in all tested cells. The cellular uptake, intracellular ROS generation and, consequently, overall photodynamic activity of F_2_POH was significantly enhanced after its incorporation in polymeric micelles of triblock copolymer (L121). *In vitro* PDI experiments showed that halogenated porphyrins display diverse antimicrobial efficacy after a short period of incubation and irradiation with light dose at 10 J/cm^2^. As expected, due to the nature of the envelope of Gram-negative bacteria, it was more difficult to inactivate *E*. *coli* than Gram-positive species. Susceptibility of *C*. *albicans* was intermediate between Gram-positive and Gram-negative bacteria. It was shown, that sulfonic acid derivatives very efficiently inactivate *Staphyloccocus aureus* in PBS suspensions because the peripheral sulfonic (-SO_3_H) groups can acquire a negative charge at physiological pH. Only the sulfonamide conjugate (Cl_2_PEt) was cytotoxic against fungal yeast in the dark (1 log decrease). In contrast with the PDT efficacy obtained in human and murine cell cultures, the incorporation of the porphyrins in L121 micelles did not improve significantly the PDI efficacy. This reveals that the molecular design of tetrapyrrolic structure to improve PDT and PDI efficacies cannot be dissociated from tailored development of drug formulations. It is interesting to note that fluorinated and sulfonated porphyrin (F_2_POH) turned out to be a selective PS against *Staphyloccocus aureus* over mammalian cells when it was dissolved in PBS, but once encapsulated in L121 micelles it can effectively serve as PS in anticancer approach.

## Supporting information

S1 Fig Photostability of studied porphyrins in EtOH. Irradiation of the solutions was carried out using 75 mW xenon lamp through water filter and 550 nm cut-off filters.
(TIF)

S2 Fig Fluorescence intensity at the maximum of absorption band (ca. 700 nm) of photosensitizers (c = 5 μM) as a function of increasing (a) LDL, (b) HDL concentration.
(TIF)

S3 Fig Particle size distribution of F_2_POH-L121 (a) and Cl_2_POH-L121 (b) measured by DLS in RT in PBS solutions.
(TIF)

S4 Fig Representative data with dark cytotoxicity of F_2_POH against several cancer cell lines. (TIF)

S5 Fig PDI of *S*. *marcescens* (a) and *E*. *faecalis* (b) mediated by halogenated porphyrin derivatives. Cells were incubated with PS for 1 h and exposed (or not) to 10 J/cm^2^ of visible light (420±20 nm).
(TIF)

S6 Fig The photodynamic activity of encapsulated porphyrins with Pluronic L121 against tested microorganisms. (TIF)
