# Correction: Properties of halogenated and sulfonated porphyrins relevant for the selection of photosensitizers in anticancer and antimicrobial therapies

**DOI:** 10.1371/journal.pone.0191777

**Published:** 2018-01-19

**Authors:** Barbara Pucelik, Robert Paczyński, Grzegorz Dubin, Mariette M. Pereira, Luis G. Arnaut, Janusz M. Dąbrowski

In the Funding section, the grant number from the funder National Science Center (NCN), Poland within the Sonata Bis is listed incorrectly. The correct grant number is: 2016/22/E/NZ7/00420.

There is an error in the ninth sentence of the third paragraph in the “Photophysical and photochemical properties of halogenated porphyrins” section of the Results and discussion. The correct sentence is: However, this explains why the treatment of actinic keratosis (a lesion located less than 2 mm deeper in the epidermis) is as effective with Levulan® Kerastick® using blue light at 417 nm, as it is with Metvix® using Aktilite at 630 nm.

There is an error in the first sentence of the first paragraph in the “Investigation of PS-plasma protein interactions” section of the Results and discussion. The correct sentence is: The binding of the photosensitizers to human serum albumin (HSA) plays an important role in their biodistribution and PDT efficacy *in vivo*.

There is an error in the sixth sentence of the first paragraph in the “Photodynamic activity against cancer cells” section of the Results and discussion. The correct sentence is: 20 μM of F2POH- Pluronic L121 with light doses 10–20 J/cm2 lead to ca. 90% mortality of A549 and CT26 and 50% in case of murine endothelial cells.

There is an error in the caption for Fig 2. Please see the complete, correct Fig 2 caption here.

**Fig 2. Normalized electronic absorption (a) and fluorescence (b) spectra of fluorinated sulfonated porphyrin (F**_**2**_**POH) registered in EtOH at room temperature.** Inset in 2a shows the absorbance profile with multiplied values at λ>450 nm.

In Fig 6, the graph labels appear incorrectly. Please see the corrected Fig 6 here.

**Fig 6:**
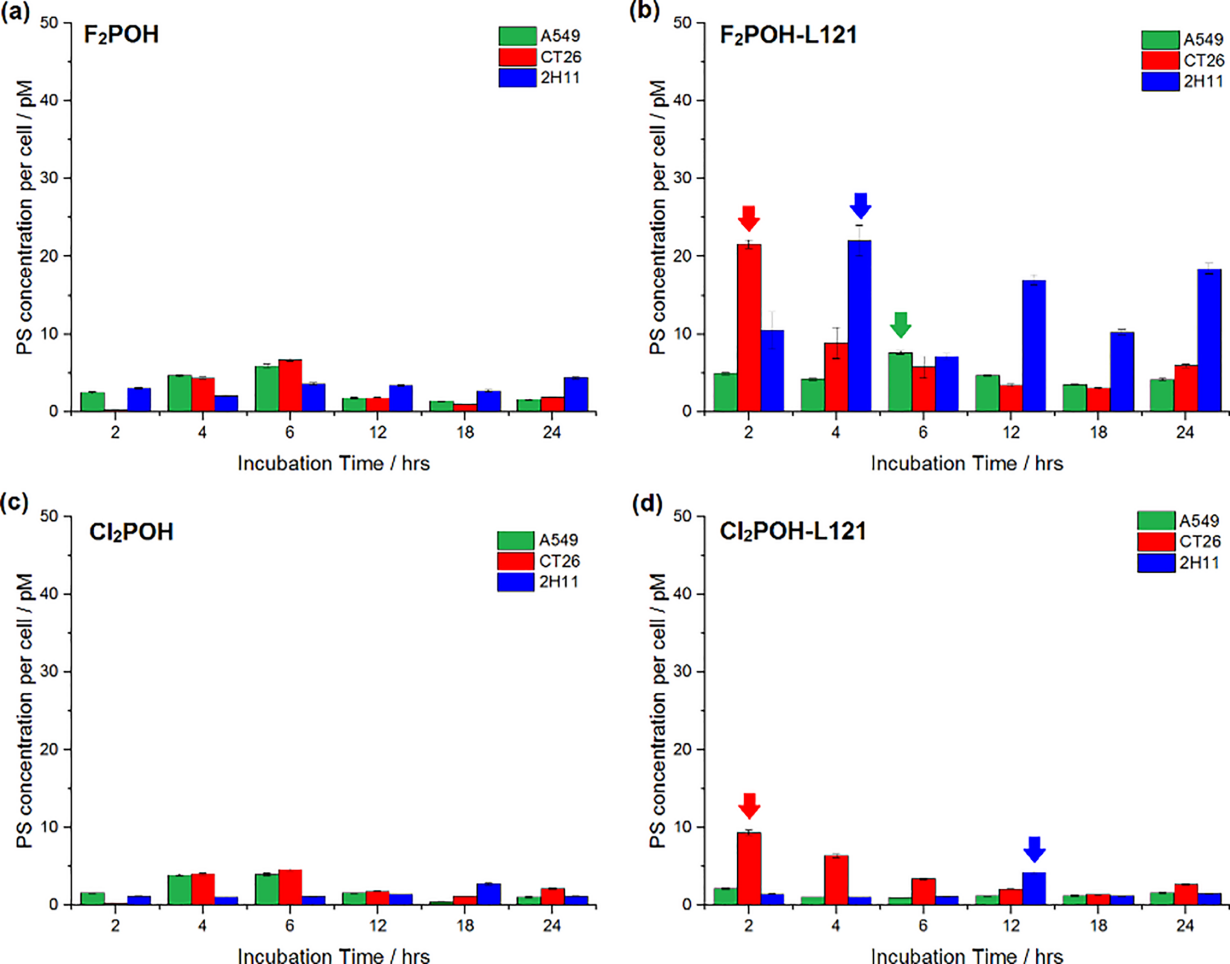
**Time-dependent uptake of hydrophilic photosensitizers (F**_**2**_**POH, Cl**_**2**_**POH) without (a, c) and with (b, d) various micellar formulations by A549, CT26 and 2H11 cells.** The data are expressed as mean value (N = 12) ±SEM.
