# Supplementary figures and images for: Properties of halogenated and sulfonated porphyrins relevant for the selection of photosensitizers in anticancer and antimicrobial therapies
Source: PLoS One. 2017 Oct 10;12(10):e0185984. doi: 10.1371/journal.pone.0185984 (PMC5634595; doi:10.1371/journal.pone.0185984)

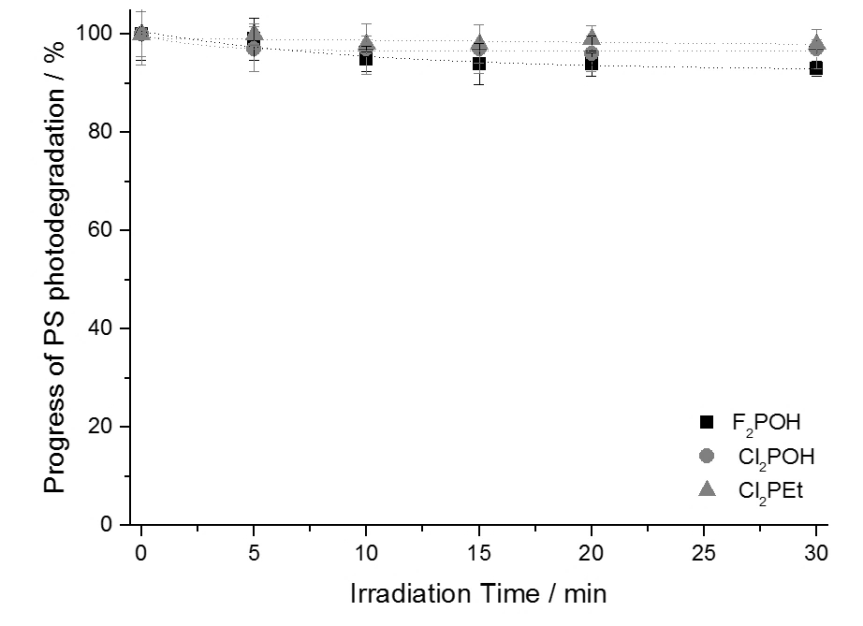

Supplement: S1 Fig — Irradiation of the solutions was carried out using 75 mW xenon lamp through water filter and 550 nm cut-off filters. (TIF) [file pone.0185984.s001.tif]

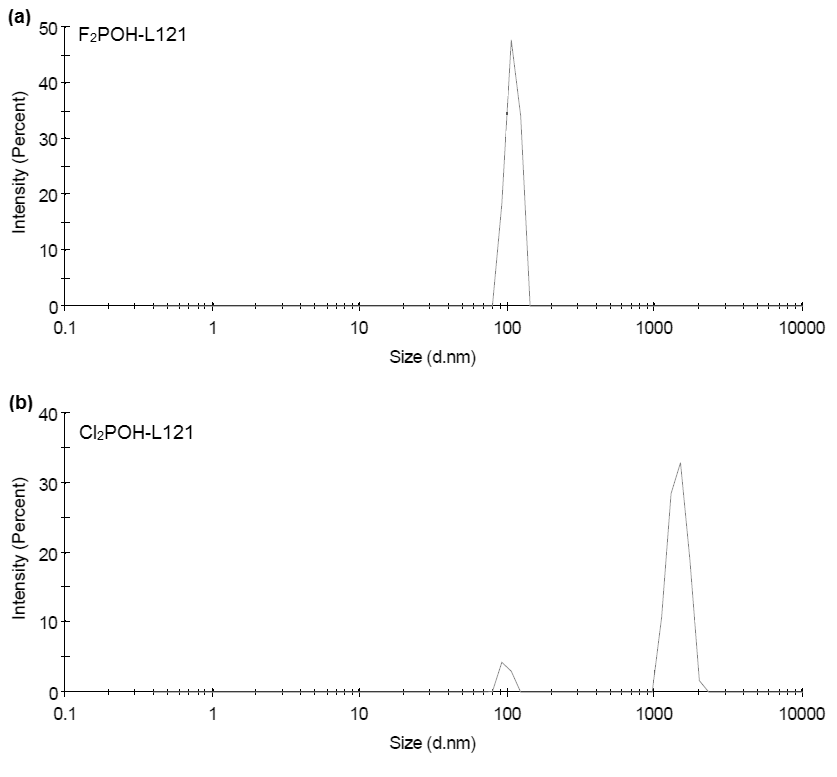

Supplement: S3 Fig — Particle size distribution of F2POH-L121 (a) and Cl2POH-L121 (b) measured by DLS in RT in PBS solutions. (TIF) [file pone.0185984.s003.tif]

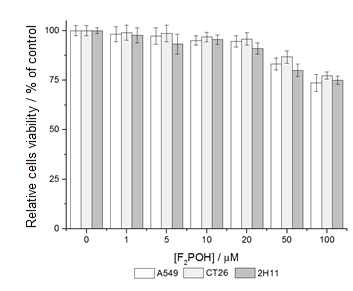

Supplement: S4 Fig — (TIF) [file pone.0185984.s004.tif]

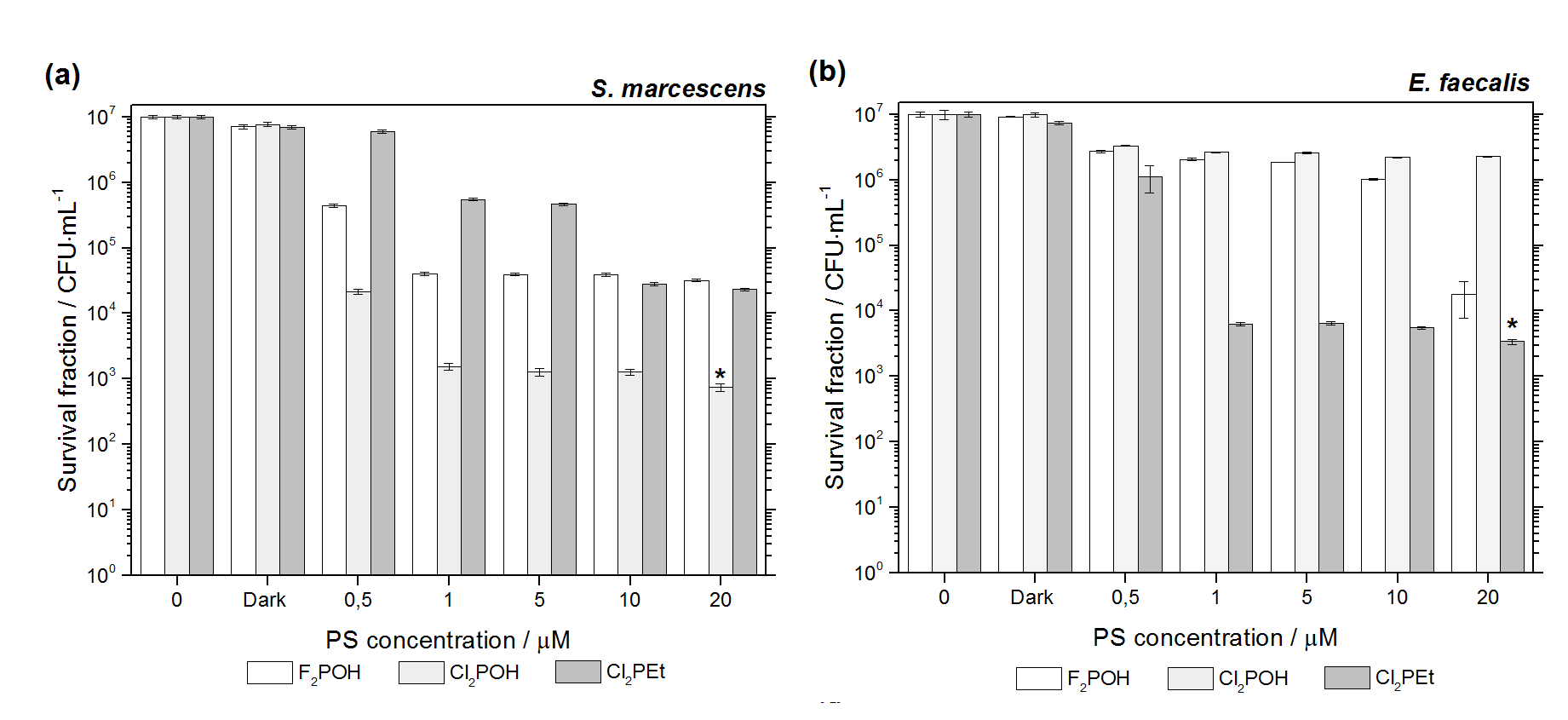

Supplement: S5 Fig — PDI of S. marcescens (a) and E. faecalis (b) mediated by halogenated porphyrin derivatives. Cells were incubated with PS for 1 h and exposed (or not) to 10 J/cm2 of visible light (420±20 nm). (TIF) [file pone.0185984.s005.tif]

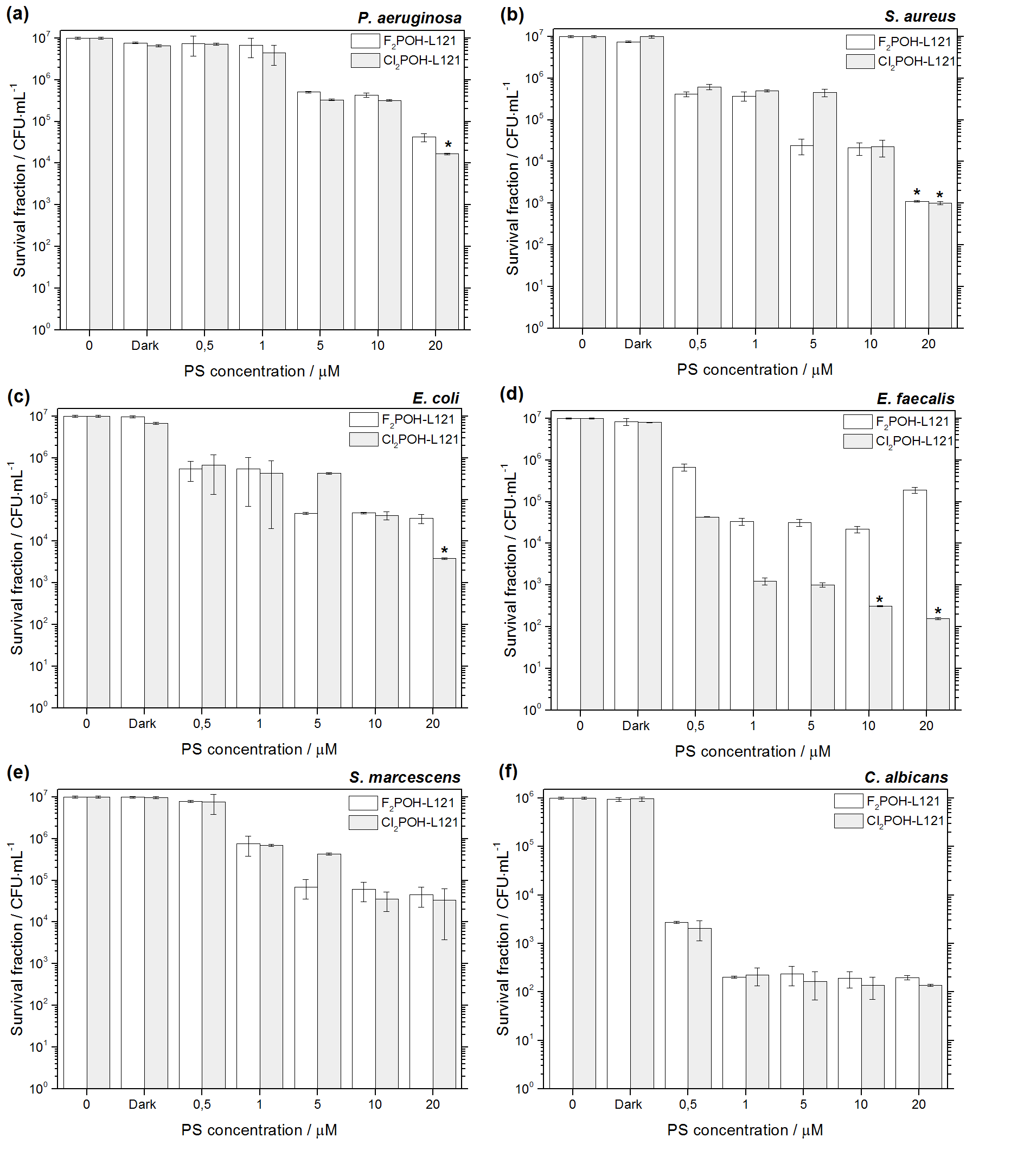

Supplement: S6 Fig — (TIF) [file pone.0185984.s006.tif]
